# Predominant Polarity for Enhanced Phenotyping and Personalized Treatment of Bipolar Disorder: A Narrative Review on Recent Findings

**DOI:** 10.1007/s11920-025-01592-x

**Published:** 2025-03-04

**Authors:** Giovanna Fico, Marta Bort, Meritxell Gonzalez-Campos, Giulia D’Alessandro, Michele De Prisco, Vincenzo Oliva, Gerard Anmella, Constanza Sommerhoff, Eduard Vieta, Andrea Murru

**Affiliations:** 1 Department of Medicine, Faculty of Medicine and Health Sciences, Institute of Neurosciences (UBNeuro), University of Barcelona (UB), C. Casanova, 143, Barcelona, Catalonia 08036 Spain; 2 Bipolar and Depressive Disorders Unit, Hospìtal Clinic de Barcelona, c. Villarroel, 170, Barcelona, Catalonia 08036 Spain; 3 Institut d’Investigacions Biomèdiques August Pi i Sunyer (IDIBAPS), c. Villarroel, 170, Barcelona, Catalonia 08036 Spain; 4 Centro de Investigación Biomédica en Red de Salud Mental (CIBERSAM), Instituto de Salud Carlos III, Madrid, Spain; 5 Psychiatric Unit, Department of Clinical and Experimental Medicine, University of Pisa, 56121 Pisa, Italy

**Keywords:** Predominant polarity, Polarity index, Bipolar disorder, Neurobiology, Subphenotype, Endophenotype

## Abstract

**Purpose of Review:**

This paper explores Predominant Polarity (PP) in Bipolar Disorder (BD), defined as the predominance of either manic or depressive episodes over a patient’s course of illness. We examine its clinical relevance, neurobiological foundations, and potential for guiding personalized treatment strategies. The review seeks to determine whether PP is a reliable course specifier and how it can be utilized to improve clinical outcomes.

**Recent Findings:**

PP has a significant impact on prognosis and treatment planning in BD. Manic and depressive PP are associated with distinct clinical and neurobiological profiles of BD, while individuals without a clear predominance of either episode type represent a more severe to-treat subgroup of patients. The development of the Polarity Index (PI) facilitates treatment decisions based on PP.

**Summary:**

PP offers a valuable framework for refining BD treatment and understanding its complexity. Future research should focus on refining PP definitions, validating neurobiological markers, and integrating these insights into comprehensive treatment models to improve patient outcomes.

## Introduction

Bipolar disorder (BD) is a severe psychiatric condition characterized by pathological mood swings ranging from mania to depression. When untreated or poorly controlled it often results in damaging personal, social, or vocational consequences, with an overall functioning impairment that persists even during inter-episode periods in a substantial portion of patients [1, 2]. BD poses a substantial challenge for both clinicians and researchers due to its inherent complexity and heterogeneity. Despite the numerous pharmacological treatment guidelines, most individuals still experience high rates of mood recurrences or significant residual symptoms while on medication [3–5]. The long-term outcomes of BD range from lasting remission to chronic disabling mood symptoms [6]. Identifying clinical sub-types associated with different long-term outcomes is essential to guide precision treatment interventions, and even pave the way for proactive and preventive strategies [7].

Recently, there has been renewed interest in the concept of predominant polarity (PP), a course specifier for BD not included in current diagnostic systems. PP appears to be a valid neurobiological endophenotype in characterizing BD and provides useful information for clinicians [8, 9]. The concept of PP was forged to define subgroups of patients with BD who mainly experience recurrences of depressive or manic episodes [10]. Over time, several definitions were operationalized to better characterize different phenotypes within the same diagnostic frame of BD. According to this definition, a depressive PP (DPP) is found in up to 35-50%, whilst a manic PP (MPP) is observed in up to 55% of individuals with BD [11]. Different PPs are linked to distinct phenotypes of BD, and their identification can help guide psychopharmacological treatment strategies [12].

Despite its widespread acceptance, the definition of PP is controversial and has faced criticism over the years. One primary concern is that the mathematical calculation of PP may not fully capture the clinical nuances of the associated phenotypes, in particular the sub-clinical episodes. Further, there are challenges in integrating mixed states and episodes into the PP framework, as these episodes do not align with the binary categorization of depressive or manic predominance. Furthermore, a significant proportion of patients with BD do not exhibit a specific PP, complicating the application of this classification. Another issue is the variability in translating findings related to PP across different cohorts, which can limit the generalizability of research outcomes.

In light of these challenges, we conducted a comprehensive review of the relevant clinical and neurobiological evidence that supports the recognition of PP as a valid course specifier of BD, to highlight the need for the implementation of PP in clinical practice, overcome common criticisms, and lay the groundwork for further exploration of these clinical specifiers in international cohorts.

## Clinical Implications of Predominant Polarity

### Conceptualization and Operationalization of Predominant Polarity

The concept of PP in BD was first described by Angst in 1978 who differentiated patients based on the predominance of manic or depressive episodes during a 16-year follow-up period, resulting in three categories: “preponderantly manic,” “preponderantly depressed,” and “nuclear,” for cases lacking a clear episode polarity predominance [10]. Over time, the definition of PP has evolved. Early approaches to defining PP were based on a simple count of lifetime episodes, distinguishing between depressive and manic predominance [13, 14]. This approach, however, lacked precision and consistency, prompting the development of more structured frameworks.

Subsequently, the need to operationalize the concept led to two widely accepted definitions. The first one, known as the *Barcelona Proposal* [15], defines MPP or DPP based on *at least two-thirds of lifetime episodes being of one polarity*. This model has been extensively validated and is widely used in clinical settings. The second one, known as the *Harvard Proposal or Index* [16], classifies patients simply according to the *majority* of episodes (i.e., > 50%) being of a particular polarity. This criterion increases the number of patients who could be categorized with a PP but did not identify significant differences in clinical correlates among the subgroups and has less diagnostic stability compared to the *Barcelona Proposal* [6, 17].

Although the Barcelona Proposal is the most consistently used and validated [9, 18], it also attracted criticism. First, it implies a restrictive definition, resulting in approximately 50% of patients [19, 20] falling into the category of undetermined PP (UPP), which is associated with a more severe and difficult-to-manage course of BD. Also, the definition of PP does not account for mixed states or episodes, and there is substantial variability in how these episodes have been included in the PP classifications in previous studies. A mixed episode, according to the DSM-IV, occurs when an individual simultaneously meets the criteria for both a major depressive episode and a manic episode nearly every day for at least one week. The DSM-5 revises this definition to a “mixed features” specifier, where symptoms of the opposite mood state are present during a manic, hypomanic, or depressive episode, without requiring the full criteria for both episodes [21, 22]. Some studies have categorized mixed episodes under MPP [8], while others have included them under DPP [23]. Alternatively, mixed episodes have been counted towards the total number of episodes without assigning them to a specific polarity [15], thereby increasing the proportion of patients classified as having an undetermined PP (UPP) [24]. These symptoms must be significant enough to cause distress or impairment in functioning. Recently, a strategy to integrate mixed episodes in the definition of PP has been proposed, suggesting an optimal cut-off of 22.8% (approximately 1 out of 4) of lifetime mixed episodes relative to the total number of affective episodes to identify patients with a distinct mixed phenotype. This subgroup was associated with higher rates of BDI diagnosis, suicide attempts, self-aggressive behavior, and a greater total number of affective episodes [25].

### Clinical Correlates of Predominant Polarity

A Task Force on the nomenclature and course of BD provided by the International Society of Bipolar Disorders (ISBD) concluded that the clinically derived PP construct developed by Angst and operationalized by Colom et al. is a valid course specifier for BD [26]. Although the clinical use of the PP concept has robust clinical evidence regarding the clinical management of BD patients [19], the DSM-5-TR did not include ‘predominant polarity’ as a course specifier for BD [27]. Most recent international guidelines take into consideration the concept of PP as a valid course specifier for BD [28–32]. Nonetheless, the utility of PP in clinical practice is supported by a substantial body of evidence, which underscores its relevance in the clinical management of BD.

**Fig. 1:**
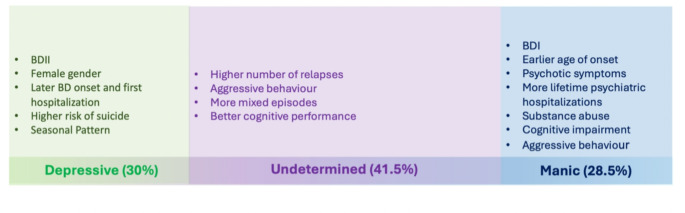
Clinical correlates of predominant polarity in BD (adapted from Colom et al., [15])

Clinical variables associated with MPP include earlier age of onset, male gender, BD-I subtype, the presence of psychotic symptoms, and index manic episodes [33]. Conversely, DPP is more common in females and is linked to index depressive episodes, a greater number of lifetime mood episodes, and a history of suicide attempts [11]. The higher prevalence of BD-II among females and its association with DPP, compared to BD-I’s association with MPP among males, may explain observed gender differences in polarity. DPP is further correlated with delayed BD diagnosis and an increased rate of comorbid anxiety disorders. MPP, on the other hand, is associated with poorer cognitive outcomes, suggesting a significant contribution of manic episodes to cognitive impairment and neuroprogression in BD [34]. Therefore, cognitive impairment could be more associated with the polarity of the episodes rather than their frequency, making PP a crucial specifier for predicting progressive cognitive impairments [35]. Substance abuse history prior to the first manic or depressive episode is more prevalent among those with MPP. Longitudinal studies demonstrate that patients with DPP experience a poorer prognosis, with more frequent depressive episodes, hospitalizations, and suicide attempts, as well as persistent substance abuse, compared to patients with MPP, who showed a reduction in substance use over time [12, 17, 36]. Recent research showed that the PP concept should be regarded as a continuum between the extremes of manic and depressive polarity, with UPP as an intermediate group. The clinical correlates of UPP have been only recently addressed in the literature [24] and include rapid cycling, suicide attempts, and psychotic symptoms, but better performances in various neuropsychological domains when compared to MPP or DPP [12]. Moreover, UPP has been linked to a higher occurrence of lifetime mixed and total affective episodes, seasonality, and aggressive behaviors [37, 38] resembling characteristics of the mixed phenotype. Given that patients with DPP or MPP might receive more specific interventions and tailored pharmacological treatments, UPP might underpin a subpopulation of patients more difficult to manage, with a higher number of relapses, also facilitated by a lower adherence to treatments, and/or rapid cycling, ultimately leading to worst outcomes (see Fig. 1). This also might be an effect of the age of the cohorts since BD often exhibits a shift in clinical presentation as individuals age, with a notable increase in the frequency of depressive episodes. In the early teenage years, the disorder typically manifests through depressive episodes, which evolve in character over time [39]. As patients enter middle age and older adulthood, depressive symptoms become more prevalent and are a stronger predictor of overall functioning compared to manic symptoms [40]. Research also indicates that the depressive polarity of episodes increases over the lifespan, with a concurrent decline in the occurrence of manic and mixed episodes [41].

### Pharmacological Treatment of Bipolar Disorder According to Predominant Polarity

PP plays a critical role in guiding therapeutic strategies for patients with BD [42]. Notable clinical differences between predominantly manic and depressive BD patients underscore the necessity for tailored pharmacological maintenance treatment based on a patient’s PP [43]. Optimal clinical practice, particularly for maintenance treatment, typically involves interventions that are more frequently guided by PP rather than by index episodes or other clinical features. The Polarity Index (PI) is a key metric used to classify maintenance therapies, distinguishing between those with a predominant antimanic prophylactic profile and those with an antidepressant prophylactic profile [42]. PI is defined as the ratio of the number needed to treat (NNT) to prevent a depressive episode to the NNT to prevent a manic episode. A PI greater than 1 indicates stronger antimanic effects, while a PI less than 1 suggests stronger antidepressant effects (Table 1). A PI of 1 reflects equal efficacy in preventing both manic and depressive episodes. For example, patients with DPP may respond better to treatments such as lamotrigine (PI < 1) and lurasidone and may require adjunctive antidepressants to manage acute bipolar depression episodes [18, 44]. Conversely, patients with MPP show greater responsiveness to atypical antipsychotics like risperidone, aripiprazole, ziprasidone, and olanzapine, which possess high PI values [42]. Quetiapine and lithium, with a PI close to 1, are effective in preventing both manic and depressive episodes, and may also be beneficial in treating patients with a mixed phenotype [45, 46]. International treatment guidelines for BD emphasize the importance of considering PP in the formulation of long-term treatment strategies [45, 47]. By tailoring treatment approaches according to PP, clinicians can enhance therapeutic outcomes and better manage the long-term course of BD.

**Table 1:** Polarity index of pharmacological and psychological maintenance treatment of bipolar disorder

Category	Treatment	PI
Predominantly Antidepressive Treatment (< 1)	Quetiapine combined with lithium/divalproex	0.8
	Oxcarbazepine combined with lithium	0.6
	Olanzapine combined with lithium/divalproex	0.5
	Divalproex	0.5
	Lamotrigine	0.4
	Psychoeducation	0.73, 0.78
	Cognitive-behavioral therapy	0.33
	Family-focused therapy	0.42
Both Antimanic and Antidepressive (≈ 1)	Lithium	1.4
	Quetiapine monotherapy	1.4
	Enhanced relapse prevention	1.0
Predominantly Antimanic Treatment (> 1)	Paliperidone	15
	Risperidone Monotherapy	∼14
	Aripiprazole Monotherapy	10.4
	Risperidone LAI	9.1
	Aripiprazole adjunctive to lithium/divalproex	4.2
	Olanzapine monotherapy	4.0
	Ziprasidone adjunctive to lithium/divalproex	3.9
	Cariprazine	2.91
	Asenapine	∼3
	Caregiver group psychoeducation	1.78
	Brief technique-driven interventions	3.36

**PI = Polarity index**: A measure of the relative efficacy of treatments for depressive versus manic symptoms. **<1**: Predominantly antidepressive treatment (more effective for depressive symptoms); **≈1**: Both antidepressive and antimanic (balanced efficacy for both depressive and manic symptoms); **>1**: Predominantly antimanic treatment (more effective for manic symptoms). Adapted from Popovic et al. 2012, 2014. and Vieta et al. 2018, 2024 [42, 48, 49]

### Non-Pharmacological Treatment and Predominant Polarity

The effectiveness of non-pharmacological treatments can also differ based on whether the PP is manic or depressive. MPP is linked to a higher likelihood of needing electroconvulsive therapy [12, 50]. Psychological interventions that emphasize cognitive engagement and face-to-face interactions with the patient tend to be more effective in preventing depressive episodes [48]. Conversely, interventions that focus on education or are directed at relatives are better suited for early detection of manic warning signs and managing stressors that could precipitate manic episodes. When properly trained, relatives can play a crucial role in preventing mania, potentially more so than depression. Nonetheless, additional research is required to pinpoint the specific psychological strategies that are most effective in preventing both depressive and manic episodes.

## Neurobiology of Predominant Polarity

While PP and onset polarity have significant clinical and treatment implications in BD, their neurobiological bases remain largely unknown. Recent advances in neuroimaging, genetics, and neurochemical research have begun to unravel the distinct neural circuits and molecular pathways that underlie different PP. By examining the differences in brain structure, function, and neurochemical activity associated with depressive and manic episodes, researchers aim to identify biomarkers and therapeutic targets specific to each polarity.

### The Genetic Underpinnings of Predominant Polarity

Recent studies have explored the genetic underpinnings of PP, focusing on specific single nucleotide polymorphisms (SNPs) and their associations with neuroplasticity, circadian rhythms, and the monoaminergic system, or exploring the role of polygenic risk scores (PRS) for major psychiatric disorders in PP.

One study investigated the genetic basis of PP by analyzing the effects of 66 SNPs associated with neuroplasticity (BDNF, ST8SIA2), second messenger cascades (GSK3B, MAPK1, CREB1), circadian rhythms (RORA), transcription (SP4, ZNF804A), and the monoaminergic system (HTR2A, COMT). Two SNPs in the ST8SIA2 gene (rs2657340 and rs4777989) were found to be associated with PP [51]. The rs2657340 polymorphism impacts neural plasticity, remodeling, and the regulation of ion channels and neurologically active molecules (BDNF, FGF2, dopamine). The rs4777989 polymorphism, associated with the VIPR2 gene, is involved in circadian rhythm regulation and has implications for BD pathogenesis. Although the functional implications of ST8SIA2 and VIPR2 are not fully understood, it is known that both influence neuroplasticity, the dopaminergic system, and circadian rhythms, indicating a potential role in BD. Another study investigated associations between polygenic liabilities (PRS) for BD, major depressive disorder (MDD), and schizophrenia and episode polarity among individuals with BD. Risk alleles for those with a manic PP are most shared with risk alleles for BD, except for psychotic mania, which shares, genetic liability with schizophrenia. Also, individuals with depressive PP or a predominance of mixed episodes share alleles most closely with those for MDD [52]. These preliminary findings lay crucial groundwork for future research but also emphasize the need for caution in drawing conclusions. The multifaceted nature of BD and its genetic architecture also shared with other psychiatric disorders [53], clearly suggests that a singular focus on SNPs or PRS does not begin to capture the disorder’s complexity. Future studies should aim to integrate genetic data with broader phenotypic and environmental factors, employing large, diverse cohorts to validate and refine these associations.

### Neuroimaging of Predominant Polarity

Numerous studies have evaluated neuroanatomical aspects in patients with BD using structural and functional MRI, yielding diverse results. These studies have shown changes in brain structures and activation patterns depending on the type of affective episode. In one study assessing differences in hippocampal subfield volumes in 175 BD patients compared to 150 healthy controls, patients with DPP and UPP exhibited global reductions in hippocampal subfield volumes, while no differences were observed in patients with MPP [54]. Specifically, patients with DPP had smaller bilateral presubiculum/subiculum volumes than those with MPP, suggesting that volume reduction could be a marker of disease progression or a risk marker for DPP and UPP.

Indeed, in a recent study on euthymic patients with BD, cortical thickness was affected most by depressive PP and least by manic PP [55].

Another study with 77 BDI patients found significant reductions in the thickness of the right fusiform gyrus, the left lingual gyrus, and the volume of the right thalamus in patients with MPP, although the absence of a healthy control group and limited sample size rendered these findings inconclusive [56]. A recent study identified significant differences in cortical thickness in regions such as the right medial frontal cortex and the left inferior frontal gyrus among others, with DPP patients showing the greatest reductions, MPP patients showing lesser reductions, and UPP patients exhibiting intermediate reductions [55].

The heterogeneity of findings across studies highlights the complexity and incomplete understanding of the relationship between brain structure and PP in BD. Despite these inconsistencies, cortical thickness appears to hold promise for identifying the neuroanatomical bases of PP, offering potentially valuable insights into anatomical alterations at the macrostructural level.

## Current Critical Points and Future Directions

Given the robust evidence supporting the validity of PP as a course specifier and its neurobiological underpinnings, there is a compelling argument for its inclusion in the DSM-5. This inclusion would provide clinicians with a standardized tool for assessing and managing BD, offering a better understanding of the illness and aiding in the development of personalized treatment strategies.

The utility of PP is evident in its capacity to categorize patients into more precise subtypes, which has significant implications for clinical management. Patients with DPP are typically at a higher risk for recurrent depressive episodes, anxiety disorders, and suicidality. In contrast, those with MPP often present with earlier onset, a predominance of psychotic symptoms, more substance-use disorders, and other unique clinical challenges. Recognizing these distinctions is crucial for developing targeted treatment plans, including the selection of pharmacological agents and the implementation of specific psychosocial interventions.

The PI exemplifies a practical application of PP in clinical settings, guiding the choice of maintenance therapies that align with a patient’s specific needs. This tool helps clinicians tailor pharmacological treatments based on the individual’s predominant episode type, potentially improving treatment outcomes [48]. Additionally, non-pharmacological interventions, such as targeted psychotherapies, play a crucial role in managing BD and should be considered in light of the patient’s PP. These therapies can be particularly effective in addressing functional impairments, enhancing cognitive outcomes, and promoting overall well-being. It is essential to study these new interventions in relation to different PP, as they offer valuable benefits that extend beyond what medication alone can achieve [57, 58]. Furthermore, with ongoing clinical trials exploring new drugs for BD that are not yet approved, there is an opportunity to calculate and apply the PI to these emerging treatments, potentially refining personalized care even further [59].

Current research is addressing criticisms related to PP. A new sub-phenotype, characterized by the absence of a specific PP (UPP), has been further detailed and described [24]. Additionally, efforts have been made to incorporate mixed episodes into the definition of PP [46]. However, concerns persist. Indeed, most studies have been conducted in specialized centers that contributed to developing an operational definition of PP, potentially introducing biases and limiting the generalizability of findings. Therefore, expanding research to include diverse global cohorts is essential to address these limitations and enhance the understanding of PP’s clinical relevance across different populations.

At the subclinical level there is often considerable variability in mood states that do not reach the level of formal clinical concern or the individual does not recognize (or acknowledge the episode) or chooses not to bring the episode to the attention of the care providers. There significant variation in symptom severity profiles over time that essentially challenge the concept of an “episode” [60]. A further challenge to the concept is the potential for successful treatment to mask or modify the phenotype, the ´wild-type’ bipolar phenotype is rarely seen in the era of modern psychiatry. The high rates of recurrence despite treatment is difficult to reconcile. Longitudinal studies that span the lifetime of the persons with bipolar are needed.

Our center contributes to this endeavor through participation in both collaborative national initiatives [61] and international efforts like the Global Bipolar Cohort (GBC) [62]. The GBC seeks to uncover the underlying mechanisms and outcomes of BD, with a focus on genetics, biological processes, and clinical care. It emphasizes the importance of large, longitudinal, and well-characterized cohorts that encompass diverse ethnicities, ensuring that research findings are applicable across various demographic contexts. As part of the GBC, we have engaged in several studies utilizing our follow-up cohorts of individuals with BD [63]. This collaborative initiative allows for the pooling of extensive data from independent studies, thereby enabling researchers to identify commonalities and divergences in clinical variables and pharmacological treatments among well-characterized BD patients worldwide. The advantage is the global nature of the study and capturing data from a wider age range of participants.

International collaboration is particularly crucial for exploring differences in PP across different cohorts, as it facilitates the examination of patterns and variations that may be influenced by genetic, environmental, or sociocultural factors. Such collaboration not only enhances the statistical power necessary to detect subtle differences but also ensures that the findings are robust and widely applicable.

PP is also a valuable concept for overcoming the limitations of traditional nosological classifications, which sometimes fail to capture the complexity of BD. It offers a more precise way to phenotype patients, enabling a deeper understanding of individual differences within the disorder.

Another significant challenge in PP research is the scarcity of extensive longitudinal studies, which are crucial for understanding the stability of PP over time [64]. Many existing studies have limited follow-up periods or are constrained by funding, making it challenging to draw definitive conclusions about the persistence of PP in individual patients. There is a pressing need for improved funding and strategic planning to support these long-term studies, as they will provide valuable insights into the natural course of BD and help develop more effective, personalized treatment strategies [65].

Existing evidence suggesting that PP is stable in most patients but can shift under certain conditions. Notably, transitions from MPP or DPP to UPP are more common, particularly in individuals with a higher cumulative number of affective episodes [24]. These findings suggest that the cumulative burden of episodes may destabilize PP over time, increasing rates of UPP. While the role of age in PP changes is debated, other factors such as medication adherence, comorbidities, and substance use appear to play significant roles. Exploring the predictive value of early PP, such as during the ages of 20–30 or the first decade after symptom onset, could provide crucial insights into long-term clinical outcomes and inform individualized treatment strategies.

Looking ahead, future research should go deeper into the genetic underpinnings of different phenotypes within BD, especially considering the limitations of recent genome-wide association studies (GWAS). While GWAS have enhanced our understanding of the neurobiology of BD [66], they typically use broad diagnostic categories that fail to capture the full spectrum of phenotypic variability in the disorder. A more refined definition of phenotypes could reveal distinct genetic profiles associated with each, providing valuable insights into the biological basis of these variations. Traditional diagnostic labels are insufficient for describing the multidimensional nature of behavioral disorders [67]. To address these shortcomings, several initiatives have emerged in recent years, aiming to provide a more nuanced understanding of the phenotypic spectrum in BD and beyond [68]. Additionally, many clinical correlates of PP remain unexplored, such as those related to affective cognition. Indeed, in patients with BD, emotional intelligence [69], facial emotion recognition [70], reward and punishment processing [71], and emotion regulation are often impaired [72]. Currently, there is indirect evidence of correlations between emotion regulation and PP, as various emotion regulation strategies correlate differently with the dimension of depressive symptoms and the dimension of manic symptoms, although PP is not directly [73]. Direct evidence has emerged in the context of facial emotion recognition, where euthymic patients with MPP exhibited an avoidance of happy faces, interpreted as a protection mechanism against triggers of mania [74].

By addressing these gaps, the global research community can work towards a more accurate and comprehensive understanding of BD, ultimately improving patient outcomes worldwide.

## Conclusions

The integration of PP as a course specifier of BD might represent a significant step forward in the understanding and management of this disease. The robust evidence supporting the neurobiological underpinnings and clinical utility of PP underscores its potential to enhance diagnostic precision and tailor treatment strategies. However, challenges remain in fully realizing the benefits of PP in clinical practice. The variability in mood states, the impact of successful treatment on phenotype expression, and the limitations of current research, particularly the scarcity of long-term longitudinal studies, highlight the need for continued investigation. Expanding research to diverse global cohorts and focusing on the genetic, environmental, and sociocultural factors that influence PP will be crucial in addressing these challenges. Future research should also delve deeper into the genetic and clinical correlates of PP, including affective cognition, to further enhance the specificity and effectiveness of treatment approaches.

## Data Availability

No datasets were generated or analysed during the current study.
